# Morphology and Phylogenetic Positions of Two Novel *Gogorevia* Species (Bacillariophyta) from the Han River, South Korea

**DOI:** 10.3390/plants14091272

**Published:** 2025-04-22

**Authors:** Weihan Wang, Yuyao Li, Byeong-Hun Han, Su-Ok Hwang, Baik-Ho Kim

**Affiliations:** 1 Department of Environmental Science, Hanyang University, Seoul 04763, Republic of Korea; wwhann4811@gmail.com (W.W.); liyuyao7773@163.com (Y.L.); tttyy3@gmail.com (B.-H.H.); 2 Research Institute for Natural Sciences, Hanyang University, Seoul 04763, Republic of Korea; spring3974@naver.com

**Keywords:** biodiversity, freshwater diatoms, *Gogorevia*, Han River, molecular phylogeny, raphid pennate, taxonomy, ultrastructure

## Abstract

This study reports two novel species, *Gogorevia contracta* sp. nov. and *G. recticentralis* sp. nov., which were isolated from freshwater environments in South Korea. Using an integrative taxonomic approach, we conducted morphological analyses using light microscopy and scanning electron microscopy, along with molecular phylogenetic investigations using *SSU* rRNA and *rbcL* gene sequences. Phylogenetic reconstructions highlighted the distinct characteristics of both species, confirming their classification within the genus *Gogorevia* and elucidating their evolutionary relationships. Morphologically, *G. contracta* was characterized by a bow-tie-shaped central area and circular depressions in the rapheless valve, whereas *G. recticentralis* exhibited a rectangular-to-wedge-shaped central area with parallel striae near the center of the raphe valve. Our findings highlighted the ecological significance of *Gogorevia* species and suggested their potential role as bioindicators of water quality in relatively unpolluted freshwater systems. Over the past decade, our research has focused on the taxonomic and ecological study of diatoms in the Han River system and identified 136 species, including nine newly described taxa. The findings of the present study contribute to a growing understanding of *Gogorevia* diversity, underscore the importance of region-specific diatom indices, and support the integration of morphological and molecular methods into diatom systematics.

## 1. Introduction

Diatoms (Bacillariophyta) are a diverse and ecologically significant group of microalgae that play crucial roles in aquatic ecosystems. Monoraphid diatoms are characterized by a raphe on only one valve, a trait that has evolved independently multiple times in different pennate diatom lineages [1]. Historically, the classification of monoraphid diatoms has been complex owing to their small size and subtle morphological differences. Hence, an integrative taxonomic approach that combines traditional microscopy and molecular phylogenetics is required [2,3].

The genus *Gogorevia* was established by Kulikovskiy et al. [4] to accommodate a distinct lineage of monoraphid diatoms that were previously misclassified as *Achnanthes* sensu lato. The type species, *Gogorevia rinatii*, along with *G. ovalis*, was described in 2020 based on morphological and molecular evidence [4]. Species of the genus *Gogorevia* are morphologically characterized by narrow, rectangular frustules that form short, slightly curved chainswith lanceolate to elliptical valves, asymmetrical central areas, and a well-defined stauros [4]. These characteristics distinguish *Gogorevia* from *Achnanthidium* and *Lemnicola*, as the three genera exhibit differences in their striae arrangement, valve structure, and raphe morphology [5].

Currently, the genus *Gogorevia* comprises at least 11 recognized species, including *G. rinatii*, *G. ovalis*, *G. uniseriata*, *G. rostellata*, *G. exilis*, and *G. angustirostrata* [4,5,6,7]. Most of these species have been described from geographically restricted regions, particularly Vietnam and Africa. While species in this genus inhabit freshwater environments with varying ecological conditions, suggesting their potential roles as bioindicators of environmental stability and water quality [8]—their global biogeographic distribution remains poorly understood. The discovery of two novel *Gogorevia* species in urban streams of South Korea provides important insights into the genus’s broader geographic range and ecological adaptability. Moreover, the continued lack of representation of *Gogorevia* in global diatom phylogenetic frameworks underscores the need for further taxonomic investigations to clarify their evolutionary relationships.

Over the past decade, our research team has focused on the taxonomic and ecological investigations of diatoms in the Han River system in South Korea. Through extensive sampling and species isolation efforts, 136 diatom species have been successfully identified, nine of which have been reported as new to science [5,9,10,11]. This ongoing research underscores the rich biodiversity of diatoms in South Korea and highlights the need for precise taxonomic classification to understand their ecological significance and role in aquatic ecosystem health. Our studies have contributed to the refinement of diatom-based bioindicators for freshwater monitoring, emphasizing the importance of region-specific diatom indices.

Traditionally, diatom taxonomy has relied on morphological identification using light microscopy, with seminal studies, such as Hustedt’s taxonomic key [12], serving as a fundamental reference [13]. However, advances in scanning electron microscopy (SEM) have enabled the high-resolution imaging of diatom frustule ultrastructure, allowing for improved species differentiation [14]. Despite these advancements, SEM alone is insufficient to resolve the cryptic diversity in diatoms, resulting in the widespread adoption of molecular tools, such as small subunit ribosomal RNA (*SSU* rRNA) and ribulose-1,5-bisphosphate carboxylase/oxygenase (*rbcL*) for phylogenetic classification [15,16]. These markers have proven particularly effective in elucidating evolutionary relationships among closely related diatom taxa [17,18].

In this study, we aimed to overcome the limitations of traditional identification methods using a polyphasic approach that integrated both morphological and molecular data [19]. In other words, we aimed to refine the classification of Genus *Gogorevia* and contribute to a broader understanding of monoraphid diatom diversity. We also aimed to assess the ecological significance of these newly described species by analyzing their habitat conditions, further supporting their potential as bioindicators for freshwater quality assessment.

## 2. Results

### 2.1. Species Description

#### 2.1.1. Taxonomic Characteristics of *G. contracta* W. Wang and B.H. Kim sp. nov. (LM, Figure 1; SEM, Figure 2)

**Figure 1:**
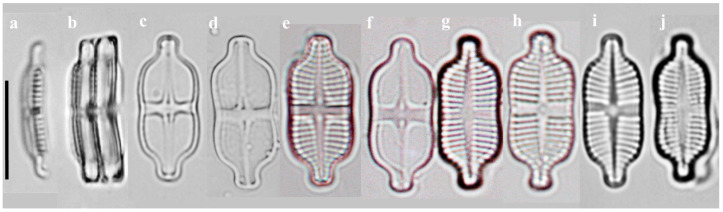
Images of *G. contracta* sp. nov. as observed under light microscope. (**a**,**b**) Girdle view; (**c**–**f**) raphe valve; (**g**–**j**) rapheless valve. Scale bar = 10 μm. The holotype specimen is shown in (**c**).

Description:

LM observations: Valves elongated and rectangular with rounded ends (Figure 1). Length 14.26–16.15 µm, width 5.25–8.28 µm and L/W ratio 1.75–2.74. Axial area narrow and linear, widening abruptly into a bowtie-shaped central area. Striae radiate on both valves, becoming more strongly radiate toward the apices. Under LM, striae generally visible (Figure 1e,g–j), although in some cases, they are nearly indistinct (Figure 1c,d,f). On the raphe valve, 35–40 striae observed in 10 µm, whereas on the rapheless valve, 15–22.5 striae present in the same distance. Areolae not resolved under LM.

**Figure 2:**
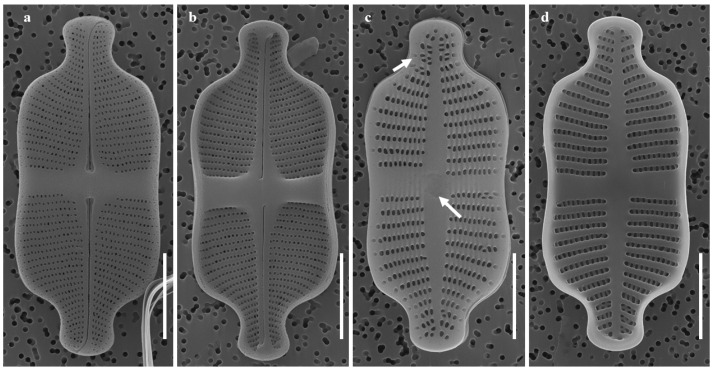

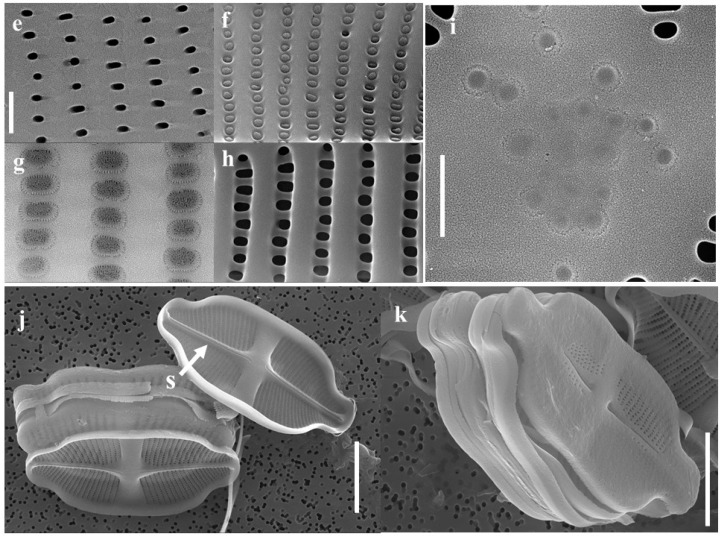
Images of *G. contracta* sp. nov. as observed under scanning electron microscope. (**a**) External view of raphe valve. (**b**) Internal view of raphe valve. (**c**) External view of rapheless valve. (**d**) Internal view of rapheless valve. (**e**) External view of areolae on raphe valve. (**f**) Internal view of areolae on raphe valve. (**g**) External view of areolae on rapheless valve. (**h**) Internal view of areolae on rapheless valve. (**i**) Central area of the rapheless valve has nearly equally sized but unevenly distributed circular depressions. (**j**) Raised central and axial stauros (S). (**k**) Girdle view. Scale bars: (**a**–**d**) = 5 µm, (**e**–**h**) = 1 μm, (**i**) = 500 nm, (**j**) = 5 μm, (**k**) = 4 μm.

SEM observations:

Raphe Valve: Raphe straight with drop-shaped central pores (Figure 2a). Central nodule externally deflected in one direction and internally reversed (Figure 2a). Terminal raphe endings oppositely oriented and deflected, terminating at valve margin (Figure 2a). Axial area and central stauros raised internally (Figure 2a,b,j). Striae uniseriate. Areolae small, elongated to rounded, and 50–60 observed in 10 µm (Figure 2e,f).

Rapheless Valve: Axial area lanceolate and narrow (Figure 2c,d). Central area asymmetric, rectangular, and had a unilateral extra stria (Figure 2c). Striae primarily uniseriate but irregularly biseriate near the center and apices (Figure 2c). Areolae elongated to rounded, and 40–50 observed in 10 µm (Figure 2g,h). Areolae on the rapheless valve differ markedly from those on the raphe valve in both size and density (Figure 2e,g). External central area nearly equal-sized, unevenly distributed circular depressions (Figure 2c,i).

Holotype: Strain HYU- D119, preserved as a permanent slide at KCTC under deposit number AG61355. The holotype specimen is illustrated in Figure 1c.

Isotype: Fixed material with permanent slides deposited at Hanyang University (Slide numbers HYU-D120 and HYU-D121).

Location: Yangjaecheon, 37°29′13.56″ N, 127°3′20.84″ E, Seoul, Republic of Korea (31 January 2024).

Etymology: The specific epithet contracta is derived from the Latin word contractus, meaning “drawn together” or “tightened”, referring to the narrowing or constriction observed in the central valve region.

Habitat: Free living on rocky substrates. Water quality parameters: WT10.69 °C; DO 13.26 mg/L; pH 7.66; conductivity 478 µS/cm; turbidity 7.0 NTU.

Molecular characterization: *SSU* rRNA (PQ046509, PQ046510, and PQ046511) and *rbcL* (PQ040372, PQ040373, and PQ040374) sequences derived from multiple isolates of *G. contracta* are available in GenBank. All sequences are identical, supporting their assignment to the same species. The *SSU* (PQ046509) and *rbcL* (PQ040372) sequences were obtained from holotype strain HYU-D119.

#### 2.1.2. Taxonomic Characteristics of *G. recticentralis* W. Wang and B.H. Kim sp. nov. (LM, Figure 3; SEM, Figure 4)

**Figure 3:**
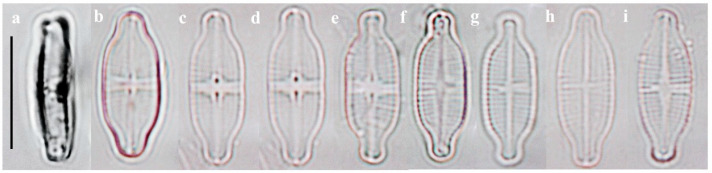
Images of *G. recticentralis* sp. nov. as observed under light microscope. (**a**) Girdle view; (**b**–**i**) valve view; (**b**–**e**) raphe valve; (**f**–**i**) rapheless valve. Scale bar = 10 μm. The holotype specimen is shown in (**b**).

Description:

LM observations: Valve length 13.04–14.65 μm, width 4.86–5.89 μm, and L/W ratio 2.45–2.85. Valves capitated with parallel sides (Figure 3). Axial area very narrow and linear, widening abruptly into a symmetrical, rectangular-to-wedge-shaped central area. Striae microradiate to parallel on the raphe valve and parallel to the microradiate on the rapheless valve (Figure 3). Raphe and rapheless valves have similar striae density, with 25–28 in 10 µm. Striae slightly visible under LM (Figure 3). Areolae not resolved under LM.

**Figure 4:**
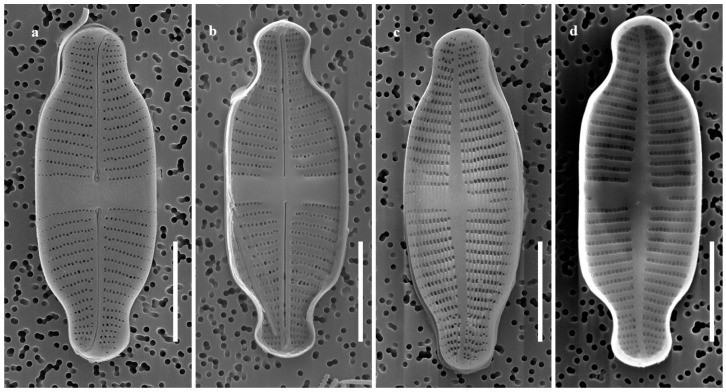

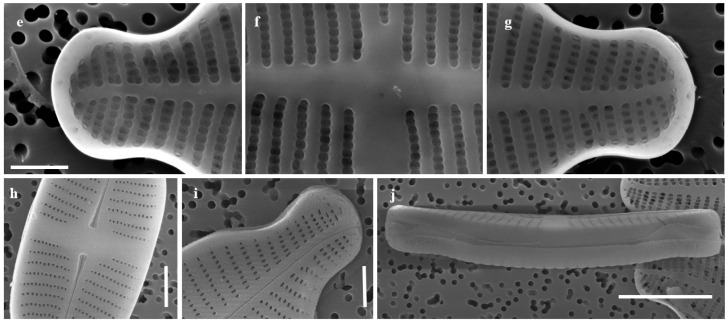
Images of *G. recticentralis* sp. nov. as observed under scanning electron microscope. (**a**) External view of raphe valve; (**b**) internal view of raphe valve; (**c**) external view of rapheless valve; (**d**) internal view of rapheless valve; (**e**,**g**) end of internal rapheless valve, with regularly distributed pore-like structures; (**f**) central area of internal rapheless valve; (**h**,**i**) external view of raphe valve. (**j**) Girdle view. Scale bars: (**a**–**d**) = 5 µm, (**e**–**g**) = 1 µm, (**h**–**i**) = 2 µm, (**j**) = 4 µm.

SEM observations:

Raphe Valve: Raphe straight with drop-shaped proximal endings. Distal raphe endings were deflected and terminated at the valve margins (Figure 4a,b). Axial area and central stauros slightly raised internally (Figure 4b). Striae uniseriate, occasionally biseriate near the axial area toward the ends (Figure 4a). Areolae elongated to rounded and occluded by hymenes (Figure 4a,h,i); 65–70 areolae observed in 10 μm, with the same density as the rapheless valve.

Rapheless Valve: Axial area slightly deflected at the ends (Figure 4c). Striae predominantly uniseriate but become irregularly biseriate near the central and apical regions (Figure 4c). Central area asymmetrical and wedge-shaped, with one side having shortened striae and slight internal elevation (Figure 4c,e–g). Areolae densities matched those of the raphe valve (65–70 in 10 μm). Areolae elongated to rounded (Figure 4c).

Holotype: Strain HYU-D123 preserved as a permanent slide at KCTC under deposit number AG61359. The holotype specimen is illustrated in Figure 3b.

Isotype: Fixed material with permanent slides deposited at Hanyang University (slide numbers HYU-D125 and HYU-D126).

Location: Godugyo, 37°51′16.04″ N, 127°44′52.09″ E, Chuncheon, Republic of Korea (18 August 2023).

Etymology: The specific epithet recticentralis is derived from the Latin words rectangularis and centralis, meaning “rectangular” and “central”, respectively, and referring to the distinctly rectangular central area of the valve.

Habitat: Free living on rocky substrates. Water quality parameters: WT 23.74 °C; DO 9.42 mg/L; pH 7.25; conductivity 156 µS/cm; turbidity 10.5 NTU.

Molecular Characterization: *SSU* rRNA (PQ034673, PQ158273, and PQ158274) and *rbcL* (PQ040372, PQ040373, and PQ040374) sequences derived from multiple isolates of *G. recticentralis* available in GenBank. All sequences are identical, supporting their assignment to the same species. The *SSU* (PQ034673) and *rbcL* (PQ040372) sequences were obtained from holotype strain HYU-D123.

### 2.2. Comparative Morphology

Table 1 presents a comparative analysis of *G. contracta* and *G. recticentralis* with morphologically similar species, including *G. exilis* [20,21], *G. rinatii* [4], *G. uniseriata* [4,5], *G. rostellata* [4,21,22], and *G. australexiguum* [21] (Table 1; Figure 1 and Figure 2 for *G. contracta*; Figure 3 and Figure 4 for *G. recticentralis*).

Valve shape, striae density, and striation are the key differentiating factors among these species. *G. exilis* exhibited a linear–elliptic to elliptic–lanceolate valve with narrowly capitated apices and radiated striae on both valves, transitioning to a near-parallel arrangement at the apices. *G. rinatii* had a lanceolate to elliptical valve with a rostrate shape and uniseriate, radiated striae that became biseriate near the axial area. *G. uniseriata* featured a concave raphe valve, whereas *G. rostellata* had a linear–elliptical shape with subcapitate apices and nearly parallel striae at the rapheless valve center, becoming increasingly radiated near the apices. The valve shape of *G. australexiguum* is parallel-sided in the central area, with margins that are occasionally slightly expanded. The striae are radiate throughout the raphe valve, becoming more strongly radiate near the apices; on the rapheless valve, they are parallel in the central area and become radiate towards the apices.

In terms of raphe valve morphology, *G. exilis* had straight raphe endings that curved oppositely at the apices, with pinhole depressions at the proximal ends. *G. rinatii* possessed drop-shaped proximal raphe endings, whereas *G. uniseriata* exhibited teardrop-shaped central raphe pores accompanied by ridges. *G. rostellata* also featured teardrop-shaped proximal raphe ends, but they curved in opposite directions at the terminal fissures. *G. australexiguum* displays clearly expanded proximal raphe endings. Variations in the axial and central areas were also observed; *G. exilis* had a narrow, slightly sigmoid axial area with a wider fascia on one side, while *G. rinatii* presented a rectangular-to-wedge-shaped fascia. *G. uniseriata* possesses a wide asymmetrical bowtie-shaped structure, whereas *G. rostellata* exhibited a bowtie-shaped structure bordered by shortened striae. Notably, the feature of shallow depressions occasionally present in the central area, described for *G. australexiguum*, was also observed in *G. contracta.*

Striae density varied significantly among species. In *G. exilis* and *G. uniseriata*, striae densities were consistent between the raphe and rapheless valves. In contrast, *G. rinatii*, *G. rostellata*, and *G. australexiguum* exhibited slightly lower striae density on the rapheless valve compared to the raphe valve. Differences in the axial areas of the rapheless valve were also evident; *G. rinatii* had a sigmoid axial area, whereas *G. rostellata* exhibited a gradually widening irregular transverse area. The central areas were distinctively shaped, ranging from a small, transapically rectangular area in *G. exilis* to an asymmetrical wedge shape in *G. rinatii* and a reduced central area in *G. uniseriata*. In *G. australexiguum*, the axial area is narrow and opens rather abruptly into the central area.

Compared to these species, *G. contracta* and *G. recticentralis* displayed a unique combination of morphological features. *G. contracta* exhibited a distinct central constriction of the valve, which distinguishes it from related species that possess parallel or slightly expanded margins in the central area (Figure 1 and Figure 2). There were also marked differences in the striae density between the raphe and rapheless valves, with each valve exhibiting a contrasting stria arrangement. In other species, the striae density between the raphe and rapheless valves is either similar or only slightly different, with the raphe valve typically showing a slightly higher density. In *G. contracta*, however, the difference is pronounced, with the raphe valve occasionally exhibiting up to twice the density of the rapheless valve—a pattern not observed in other species.

*G. recticentralis*, on the other hand, was characterized by parallel striae near the central area of the raphe valve, which contrasted with the radiating striae patterns observed in related species (Figure 3 and Figure 4). Furthermore, unlike its relatives, *G. recticentralis* lacked a prominent internal structure, and the striae densities on the raphe and rapheless valves were nearly identical. These features differentiate *G. recticentralis* from the previously described *Gogorevia* species and provide reliable morphological markers for species identification.

### 2.3. Molecular Analysis

**Figure 5:**
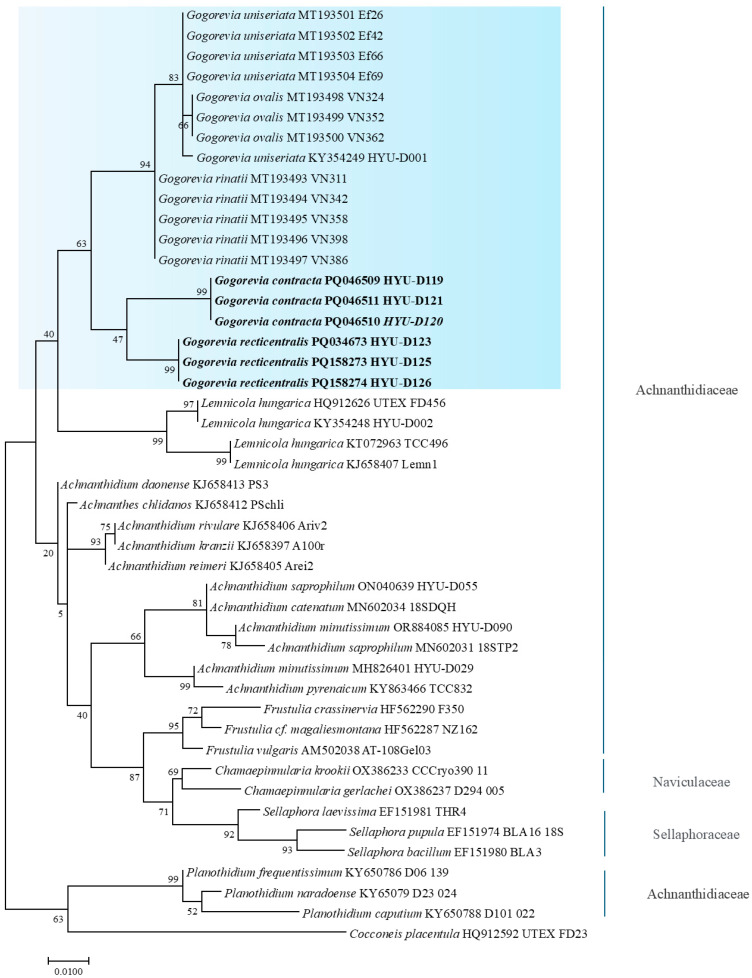
Maximum likelihood phylogenetic tree based on the *SSU* rRNA gene sequences showing the relationship of *Gogorevia* species and related monoraphid diatoms. Newly described strains from this study are shown in bold, and GenBank accession numbers are indicated. The pale blue box highlights the clade representing the genus *Gogorevia*. Bootstrap values (≥50%) based on 1000 replicates are shown at branch nodes. Scale bar indicates 0.01 substitutions per site.

**Figure 6:**
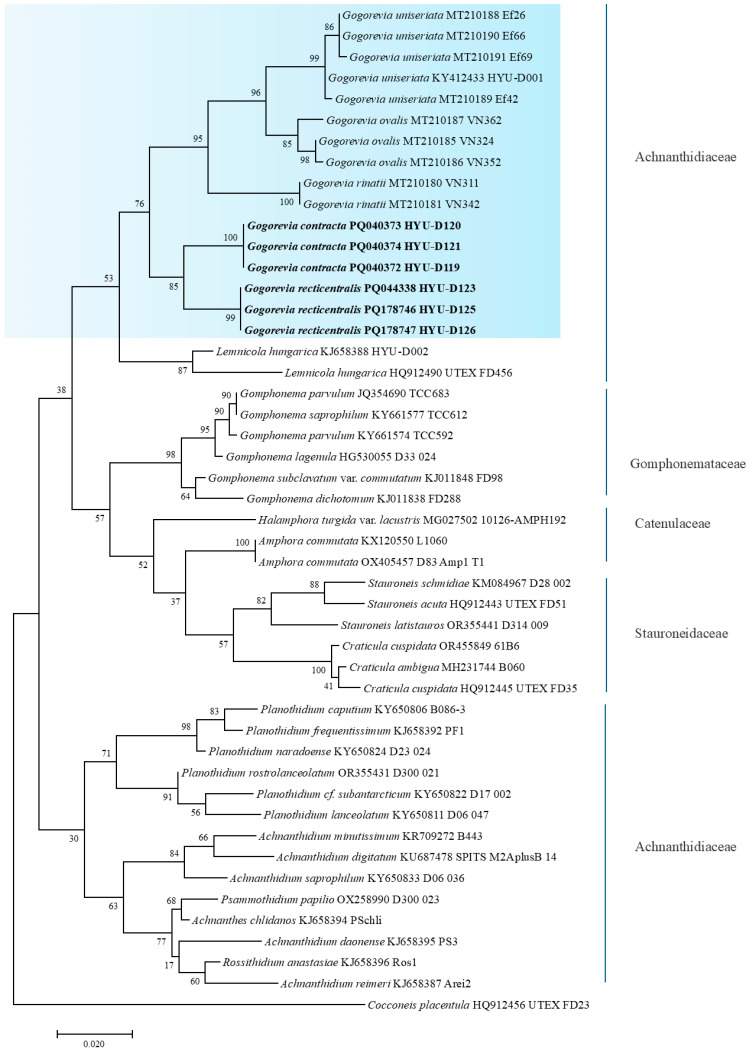
Maximum likelihood phylogenetic tree based on the *rbcL* gene sequences showing the relationship of *Gogorevia* species and related monoraphid diatoms. Newly described strains from this study are shown in bold, and GenBank accession numbers are indicated. The pale blue box highlights the clade representing the genus *Gogorevia*. Bootstrap values (≥50%) based on 1000 replicates are shown at branch nodes. The scale bar indicates 0.02 substitutions per site.

The phylogenetic positions of *G. contracta* sp. nov. (HYU–D119) and *G. recticentralis* sp. nov. (HYU–D123) were determined based on the *SSU* rRNA and *rbcL* gene sequences. Figure 5 and Figure 6 show the phylogenetic trees constructed using these markers. The analysis confirmed that *Gogorevia* species formed a distinct cluster, clearly distinguishing them from other genera within the monoraphid diatoms. A similar result was also obtained from the concatenated phylogenetic analysis, as shown in Figure S6.

Phylogenetic reconstruction indicated that *Gogorevia* and its sister genus *Lemnicola* form a well-supported monophyletic group. Within this clade, *G. contracta* and *G. recticentralis* emerged as genetically distinct lineages, which were positioned distinctly from the previously described *Gogorevia* species (Figure 5 and Figure 6). This taxonomic distinction was further supported by sequence divergence estimates.

Table 2 and Table 3 show the pairwise genetic distance values based on the *SSU* rRNA and *rbcL* sequences. The results revealed that *G. contracta* and *G. recticentralis* exhibited the smallest evolutionary divergence with pairwise distances of 0.033 (*SSU* rRNA) and 0.031 (*rbcL*), respectively (Table 2 and Table 3). In contrast, their genetic distances from other *Gogorevia* species were substantially greater, reinforcing their classification as a distinct species.

To further support the phylogenetic findings, secondary structural analyses of the *SSU* rRNA and *rbcL* sequences were conducted. Figures S1–S5 illustrate the conserved and variable regions of the molecular structures. The structural variations observed in *G. contracta* and *G. recticentralis* further differentiate them from their closest relatives, highlighting the genetic distinctiveness of the newly described species.

## 3. Discussion

### 3.1. Taxonomic and Ecological Implications of G. contracta and G. recticentralis

The discovery of *G. contracta* and *G. recticentralis* represents a significant advancement in our understanding of the morphological and genetic diversity of *Gogorevia*, a recently established genus of monoraphid diatoms [4]. The diversity and phylogenetic relationships within the genus *Gogorevia* remain insufficiently studied, with only a few species having been formally described and molecularly characterized to date. These include taxa from geographically isolated regions such as Vietnam, Africa, and South Korea. The independent discovery of *G. contracta* and *G. recticentralis* in the Han River system significantly expands the known distribution of this genus and underscores its ecological versatility in a broader range of environments [4,5,6,7]. Our study revealed that *Gogorevia* encompasses greater structural and ecological variability than previously documented, reinforcing the need for further taxonomic and ecological investigations.

Morphological analyses of *G. contracta* and *G. recticentralis* indicated distinct structural differences that distinguished them from previously described *Gogorevia* species. *G. contracta* exhibited a bowtie-shaped central area with raised stauros, whereas *G. recticentralis* possessed a rectangular to wedge-shaped central area with distinct striae orientation patterns. Moreover, *G. contracta* displayed evenly sized but irregularly distributed circular depressions on the external central area of the rapheless valve, a feature not previously documented in *Gogorevia* (Figure 1 and Figure 2 for *G. contracta*; Figure 3 and Figure 4 for *G. recticentralis*). These structural characteristics suggest that monoraphid diatoms exhibit greater morphological plasticity than was previously assumed, a pattern also observed in other naviculoid taxa [8,15].

In addition to morphological differentiation, molecular phylogenetic analyses validated these two species as distinct evolutionary entities. Phylogenetic trees constructed using *rbcL* and *SSU* rRNA sequences (Figure 5 and Figure 6) indicated that *G. contracta* and *G. recticentralis* formed separate monophyletic clades distinct from previously reported *Gogorevia* species. The phylogenetic analysis confirms that *Gogorevia* forms a monophyletic group, with *G. contracta* and *G. recticentralis* nested within the clade that includes the type species. This finding supports the original concept of the genus as a cohesive evolutionary lineage defined by both morphological and molecular characters. The observed genetic divergence was comparable to that reported in other recently classified diatom genera, further supporting the need for a broader reassessment of the genus [2,3].

Our findings highlighted the adaptive potential of *Gogorevia* species in diverse aquatic environments. *G. contracta* was identified in oxygen-rich, neutral-pH environments, whereas *G. recticentralis* was found in warmer waters with lower conductivity. These findings suggest that *Gogorevia* species may be influenced by specific hydrological and chemical conditions, reinforcing their potential use as bioindicators [12]. Given the growing role of diatoms in freshwater biomonitoring programs, future studies should assess the ecological stability of *Gogorevia* populations under changing environmental conditions [7,23].

The results of this study highlight the necessity for continued taxonomic exploration of the genus *Gogorevia*. Future research should focus on expanding the geographic sampling of *Gogorevia* species and incorporating additional molecular markers such as *ITS* and *COI* to refine phylogenetic relationships and evaluate the functional roles of these diatoms in freshwater ecosystems [24,25]. Such efforts will clarify species boundaries and enhance our understanding of diatom community dynamics in response to environmental changes.

### 3.2. Phylogenetic and Taxonomic Considerations for Gogorevia

The phylogenetic placement of *G. contracta* and *G. recticentralis* raises important questions regarding the broad classification and evolutionary trajectory of *Gogorevia*. Although the genus was initially proposed as a morphologically cohesive monoraphid taxon, emerging molecular evidence suggests that the phylogenetic analysis confirms that *Gogorevia* forms a monophyletic group, with *G. contracta* and *G. recticentralis* nested within the clade that includes the type species. This finding supports the original concept of the genus as a cohesive evolutionary lineage defined by both morphological and molecular characters.

Our findings support this hypothesis, as the phylogenetic trees based on the *rbcL* and *SSU* rRNA sequences (Figure 5 and Figure 6) revealed that *G. contracta* and *G. recticentralis* did not cluster closely with the previously described *Gogorevia* taxa. Instead, they form distinct genetic lineages, suggesting that *Gogorevia* may require taxonomic reassessment [18,25]. The significant genetic divergence observed between these species indicates that regional differentiation and ecological specialization may have played a role in their diversification, similar to the patterns observed in other pennate diatoms [24,26].

The presence of morphologically similar yet genetically distinct species within *Gogorevia* aligns with the results of previous studies demonstrating cryptic diversity within diatom genera [5]. Hence, a systematic re-evaluation of *Gogorevia* is warranted, which considers molecular, morphological, and ecological datasets. 

To refine our understanding of *Gogorevia* diversity and its evolutionary history, future research should incorporate several key approaches. First, expanding molecular phylogenetic datasets by incorporating additional markers such as *ITS* and *COI* would significantly enhance species resolution, as these markers have proven to be effective in distinguishing diatom species with subtle genetic differences [1,18]. Secondly, conducting high-resolution morphological analyses using SEM imaging would allow for a more detailed assessment of structural variations at a finer scale, thereby improving the accuracy of species delineation within a genus [27]. Finally, investigating the global biogeographic distribution of *Gogorevia* is essential for determining potential regional speciation patterns and ecological adaptations, as previous studies have demonstrated that environmental and geographic factors play crucial roles in shaping diatom diversity [20,28].

This study provides strong evidence that *G. contracta* and *G. recticentralis* are distinct from the previously described species, reinforcing the need for further taxonomic and phylogenetic research. Considering the polyphyletic nature of *Gogorevia*, continued molecular analyses and ecological assessments are essential for refining the classification and evolutionary relationships of this genus.

By integrating morphological, molecular, and ecological data, future studies could advance our understanding of diatom diversity, evolutionary processes, and biomonitoring applications in freshwater ecosystems.

In future studies, the use of “super barcodes”—including complete mitochondrial and plastid genomes, as well as full-length ribosomal DNA clusters—may provide enhanced resolution for species delimitation and evolutionary analyses in *Gogorevia* and related genera. Recent advancements in high-throughput sequencing have made such data more accessible and increasingly useful in diatom systematics.

Additionally, metabarcoding approaches using environmental DNA (eDNA) could reveal the broader biogeographic distribution of *Gogorevia* species across different freshwater habitats. Such methods hold promise for understanding the ecological preferences and regional diversity patterns of these monoraphid taxa.

## 4. Materials and Methods

### 4.1. Sample Collection and Culture

**Figure 7:**
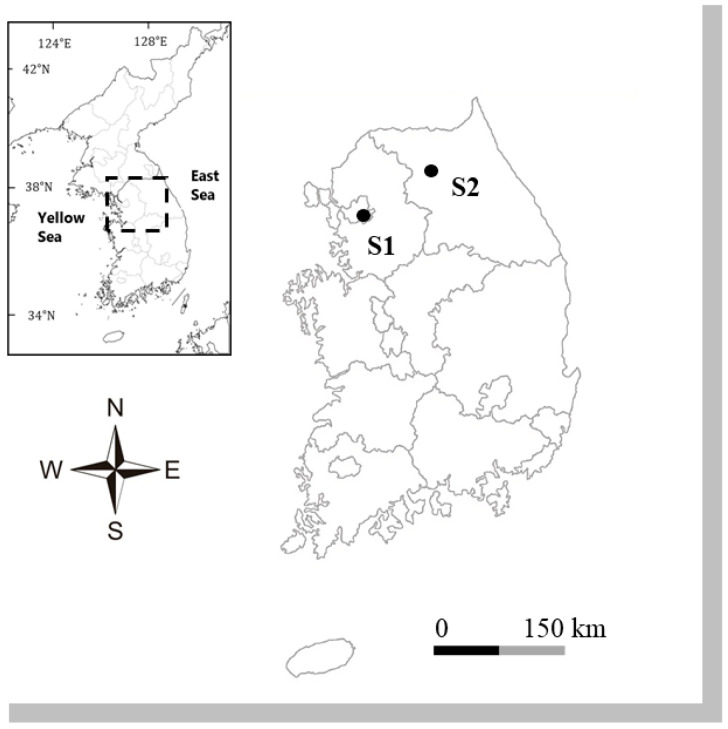
Location of sampling sites in South Korea. Site codes are given in Table 1.

Diatom samples were collected from two distinct freshwater ecosystems in South Korea as part of an ecological health assessment of epilithic diatom communities. The sampling sites included Yangjaecheon (S1) and Godugyo (S2), which represent contrasting environmental conditions and hydrological regimes and provide an opportunity to investigate species-specific ecological preferences (Figure 7; Table 4).

At each site, environmental parameters, including pH, conductivity, turbidity, dissolved oxygen (DO), and water temperature, were measured in situ using a Horiba U-50 multi-parameter water quality checker (Horiba Ltd., Kyoto, Japan), following standard protocols for freshwater quality monitoring [5]. Yangjaecheon (S1) is characterized by relatively stable hydrological conditions, with consistently high DO (13.26 mg/L) and a neutral pH (7.66). In contrast, Godugyo (S2) exhibits seasonal variations, with higher water temperatures (WT; 23.74 °C) and lower conductivity (156 µS/cm), indicative of fluctuating flow regimes. These environmental differences were analyzed to infer the ecological differentiation of species in the genus *Gogorevia*.

The sampling conditions varied seasonally. During winter, the water surface at higher latitudes was intermittently covered with ice, restricting direct access to submerged substrates. Hence, sampling was conducted from ice-free sections of the river, prioritizing areas where the epilithic diatom biofilms remained intact despite freezing. Sediment composition also differed between sites. Yangjaecheon (S1) has a stable rocky substrate, whereas Godugyo (S2) features a mixed composition of bedrock and gravel, with some areas exposed to moderate water turbulence that influenced biofilm stability. Diatom samples were collected by selecting epilithon-covered pebbles from the littoral zones at a depth of approximately 0.1 m. The biofilm was gently scraped using a sterilized silicone brush (Coplac, Tokyo, Japan) over a 5 × 5 cm surface area and transferred into sterile borosilicate glass vials (Duran Group, Wertheim, Germany) filled with filtered river water to preserve community integrity until further analysis.

Single diatom cells were individually isolated using sterilized glass capillary tubes under a CKX41 inverted phase-contrast microscope (Olympus Corporation, Tokyo, Japan) at 400× magnification. Isolation was performed under aseptic conditions to prevent microbial contamination. Successfully isolated cells were transferred into 96-well microplates (SPL Life Sciences, Pocheon, Republic of Korea) containing sterile Diatom Medium (DM), which was prepared as described by Beakes et al. [29]. Each well contained 160 μL of sterile DM, prepared with ultrapure Milli-Q water (Merck Millipore, Darmstadt, Germany) and supplemented with essential nutrients, as described by Beakes et al. [29]. Cultures were maintained in a climate-controlled incubator (DAIHAN WISE Cube, Seoul, Republic of Korea) at 20 ± 1 °C with a 12:12 h light/dark cycle under cool white fluorescent lamps (F30T8D; Panasonic Corporation, Osaka, Japan), providing 120 ± 10 μmol m^−2^ s^−1^ of photosynthetically active radiation. The light intensity was measured using an LI-250A Light Meter (LI-COR Biosciences, Lincoln, NE, USA) to ensure uniform illumination across the cultures.

After 10–14 days, when cells reached the exponential growth phase, actively dividing cultures were transferred to 50 cm³ polycarbonate culture flasks (Corning Inc., Corning, NY, USA) containing 20–25 mL of fresh DM. Cultures were regularly monitored for contamination, and only axenic cultures were selected for further subculturing.

To preserve genetic stability and physiological integrity over extended periods, selected cultures were transferred to climate-controlled storage conditions (≤10 °C) with reduced photoperiodic exposure (<20 μmol m^−2^ s^−1^) under dim LED lighting (PHILIPS Master LEDtube, Amsterdam, Netherlands). Long-term preservation was performed following established protocols for diatom culture maintenance [6,29]. Briefly, the diatom samples were fixed in acidified Lugol’s iodine solution (5% *v*/*v*; Sigma-Aldrich, St. Louis, MO, USA) and stored at 4 °C in amber glass vials (DWK Life Sciences, Wertheim, Germany) to prevent light degradation. Subsequently, the fixed samples were used for morphological, molecular, and ecological analyses.

For frustule cleaning and diatom skeletal preparation, cells were subjected to an acid digestion process using a 1:3 mixture of concentrated nitric acid (HNO_3_, 70%) and sulfuric acid (H_2_SO_4_, 98%) (Sigma-Aldrich, St. Louis, MO, USA). To ensure uniform temperature control, this process was performed at 90 °C for precisely 2.5 min in a digital dry bath (Thermo Fisher Scientific, Waltham, MA, USA). The reaction was halted by adding cold ultrapure water (Milli-Q; Merck Millipore, Darmstadt, Germany), and the samples were centrifuged at 600× *g* for 5 min in a Hettich ROTOFIX 32A centrifuge (Hettich, Tuttlingen, Germany). The supernatant was discarded, and the samples were washed at least three times with ultrapure distilled water (Milli-Q, Merck Millipore, Darmstadt, Germany) to eliminate residual acid [5]. The cleaned samples were dried and prepared for light microscopy and SEM.

### 4.2. Morphological Study of Diatoms

For light microscopy, cleaned diatom frustules were mounted using Wako Mount Media (Wako Pure Chemical Industries, Ltd., Osaka, Japan) on 25 × 75 mm glass slides (Matsunami Glass Ind., Ltd., Osaka, Japan) and dried at room temperature (15–25 °C) under sterile conditions. Permanent slides were examined under an Eclipse E600 light microscope (Nikon Corporation, Tokyo, Japan) equipped with a Plan Apo 100×/1.40 oil immersion objective. Digital images were captured using a cooled CCD camera XC10 (Olympus Corporation, Tokyo, Japan) and processed using ToupView 3.7 software (AmScope, Irvine, CA, USA).

Morphometric characteristics, including valve length, width, and striae density, were recorded for ≥60 cells per species using the stria counting method described by Schoeman and Archibald [30]. To minimize measurement errors, all image analyses were performed in triplicate, and individual cell dimensions were measured at multiple focal planes.

For SEM, the cleaned diatom samples were filtered through GTTP polycarbonate membranes (Millipore Filter Corporation, Cork, Ireland) and mounted onto 12 mm aluminum stubs (Ted Pella Inc., Redding, CA, USA) using double-sided conductive carbon tape (Nisshin EM Co., Ltd., Tokyo, Japan). The samples were then air-dried at 25 °C for 24 h.

To enhance the conductivity, a 5 nm layer of platinum coating was applied using an E1045 ion coater (Hitachi High-Technologies, Tokyo, Japan) in a vacuum chamber (5 Pa, 120 s exposure). SEM imaging was performed using an Apreo S SEM (Thermo Fisher Scientific Inc., Waltham, MA, USA), operating at an accelerating voltage of 5 kV and a working distance of 2.5 mm. To distinguish closely related species, structural observations focused on raphe morphology, central area modifications, and external valve ornamentation.

### 4.3. Molecular Study of Diatoms

Diatom cultures were harvested during the mid-logarithmic growth phase and transferred into 1.5 mL microtubes (Eppendorf, Hamburg, Germany) for further processing. Thereafter, the samples were centrifuged at 1000× *g* for 10 min using a ROTOFIX 32A centrifuge (Hettich, Tuttlingen, Germany) to pellet the cells. Following centrifugation, DNA was extracted using the DNeasy Plant Mini Kit (Qiagen, Valencia, CA, USA), following the manufacturer’s protocol.

For polymerase chain reaction (PCR) amplification, reaction mixtures were prepared with a final volume of 20 μL. Each mixture contained 10 μL of 2× BX-Taq Master Mix (NICSRO, Daejeon, Republic of Korea), 8 μL of template DNA, 1 μL of forward primer, and 1 μL of reverse primer. Two genetic markers were targeted for amplification, namely the small subunit ribosomal RNA (*SSU* rRNA) gene and *rbcL* gene. The *SSU* rRNA gene was amplified using the forward primer (AT18F01) with the sequence 5′-YAC CTG GTT GAT CCT GCC AGT AG-3′ and the reverse primer (AT18R01) with the sequence 5′-GCT TGA TCC TTC TGC AGG TTC ACC-3′ [31]. The *rbcL* gene was amplified using the forward primer (F3) with the sequence 5′-GCT TAC CGT GTA GAT CCA GTT CC-3′ and the reverse primer (R3) with the sequence 5′-CCT TCT AAT TTA CCA ACA ACT G-3′ [15].

PCR was performed using an iCycler thermal cycler (Bio-Rad, Hercules, CA, USA). The thermal cycling conditions for the reaction were as follows: an initial denaturation step at 94 °C for 4 min, followed by 37 cycles of amplification, which included denaturation at 94 °C for 20 s, annealing at 56 °C for 30 s, and extension at 72 °C for 50 s. A final extension step was carried out at 72 °C for 5 min to complete the reaction.

Following PCR amplification, the products were analyzed using agarose gel electrophoresis. Amplified DNA fragments were loaded onto a 1% agarose gel (Invitrogen, Carlsbad, USA) and subjected to electrophoresis at 120 V for 45 min. To visualize the DNA bands, the gel was stained with SYBR Safe DNA Gel Stain (Thermo Fisher Scientific Inc., Waltham, USA). After electrophoresis, amplicons were purified using the ExoSAP-IT™ PCR Product Cleanup Reagent (Applied Biosystems, Foster City, USA) to remove excess primers and nucleotides.

The obtained DNA sequences were assembled and analyzed using BioEdit v. 7.0.5.3 (Sequence Alignment Editor, CA, USA) [32]. Sequences were subsequently deposited in GenBank for public access. To compare the sequences with those of closely related taxa, reference sequences were retrieved from the National Center for Biotechnology Information (NCBI) database. Multiple sequence alignment was performed using ClustalW [33] in BioEdit v.7.6.2.0 (Ibis Therapeutics, Carlsbad, CA, USA).

Molecular phylogenetic analyses were conducted using MEGA version 7.0, employing the maximum likelihood method based on the Kimura 2-parameter model. Bootstrap support values were calculated using 1000 replicates to ensure statistical robustness [32].

To quantify the genetic variation between species, pairwise genetic distances were calculated for the *SSU* rRNA and *rbcL* genes using MEGA7, with standard error estimates obtained through a bootstrap procedure with 1000 replicates [34].

To further investigate genetic divergence, secondary structure analyses of the *SSU* rRNA and *rbcL* gene sequences were conducted using the Mfold web server [35]. The structural folding patterns of the two newly described species and three related *Gogorevia* species were compared, which helped identify conserved and variable regions in the sequences. These findings provided additional insights into the sequence variability and phylogenetic distinctiveness of the newly identified species.

### 4.4. Taxonomic Studies on New Species

Taxonomic identification of all diatom isolates was conducted using a comprehensive approach that integrated morphological and molecular analyses. For morphological classification, we consulted reference materials, such as the *Ecological Guidebook of Korean Diatoms* [36] and the *Picture Book and Ecology of Epilithic Diatoms in Korea Estuary* [37]. These resources provide detailed descriptions and high-resolution images of different species, aiding species identification of distinguishing features, such as valve shape and striae patterns.

For higher taxonomic classification, databases such as AlgaeBase [6] and Diatoms.org [22] were used to confirm species names, synonyms, and phylogenetic placement. These globally recognized databases ensured taxonomic accuracy and alignment with the latest diatom nomenclature updates.

The integration of morphological and molecular data allowed for the robust classification of diatom species, facilitating the differentiation of closely related taxa and supporting the discovery of novel species within the genus *Gogorevia*. Molecular phylogenetic analyses enhanced species identification, especially where morphological characteristics alone were insufficient to distinguish cryptic species.

This combined methodological approach offered valuable insights into species distribution, evolutionary relationships, and ecological significance. The taxonomic framework established in this study provides a foundation for future biomonitoring programs and conservation strategies aimed at preserving freshwater diatom communities in South Korea.

## 5. Conclusions

The discovery of *G. contracta* and *G. recticentralis* enhanced our understanding of monoraphid diatom diversity and highlighted the need for an integrative taxonomic approach. Morphological analyses revealed distinct valve structures and striae patterns that differentiated these species from previously described *Gogorevia* taxa. Phylogenetic analyses based on *SSU* rRNA and *rbcL* gene sequences confirmed that these species formed separate evolutionary lineages within *Gogorevia*, supporting their classification as distinct species.

Ecological analyses suggested that *G. contracta* and *G. recticentralis* are associated with specific environmental conditions, reinforcing their potential as bioindicators for freshwater quality assessments. Their occurrence in relatively unpolluted habitats indicates their preference for stable water conditions, making them valuable for biomonitoring programs [13].

Future studies should focus on expanding the molecular dataset using additional markers, such as *ITS* and *COI*, to further refine the phylogeny of *Gogorevia*. Additionally, investigating the global distribution and ecological responses of these species will improve our understanding of their evolutionary history and ecological roles in aquatic ecosystems. This discovery of *Gogorevia* species in South Korea, beyond their previously reported ranges in Vietnam and Africa, underscores the necessity of reevaluating the genus’s global biogeographyand ecological adaptability. Nonetheless, our findings emphasized the importance of integrative approaches in biodiversity research and freshwater conservation.

## Figures and Tables

**Table 1:** Morphological comparison of *Gogorevia* and related species (N.d. = No data).

	*G. contracta*	*G. recticentralis*	*G. exilis*	*G. rinatii*	*G. uniseriata*	*G. rostellata*	*G.australexiguum*
Length (μm)	14.26–16.15	13.04–14.65	5–17	11.5–15.0	5.5–8.0	15.1–19.8	14.7–18.8
Width (μm)	5.52–8.28	4.86–5.89	4.5–6.2	5–6	3.0–3.5	6.4–7.2	6.3–7.5
L/W ratio	1.75–2.74	2.45–2.85	N.d.	N.d.	1.5–2.5	N.d.	N.d.
Valve	Elongated, somewhat rectangular shape with rounded ends; middle part slightly constricted	Capitate, sides parallel	Linear–elliptic to elliptic–lanceolate with narrowly capitate, subcapitate, rostrate, or subrostrate apices	Lanceolate to elliptical with pro-tracted, short rostrate, with broadly rounded apices	Frustule slightly bent, raphe valve concave	Linear–elliptic with narrow subcapitate apices	Linear with parallel, clearly undulating margins and protracted, distinctly rostrate apices
Striation	Radiate on both valves	From apices to center, microradiate to parallel on raphe valve but parallel to microradiation on rapheless valve	Radiate on both valves, but almost parallel at the apices	Uniseriate, fine, radiate throughout, more strongly radiate near apices; sometimes striae become biseriate near the axial area	Radiate	Nearly parallel on the rapheless valve center but otherwise radiate, more so near apices; more closely spaced near apices; strongly radiate throughout and a bit more closely spaced near apices	Radiate throughout, more strongly radiate near the apices on raphe valve; parallel, becoming radiate towards the apices on rapheless valve
Areolae	Areolae on rapheless valve are noticeably larger than on rapheless; elongated to rounded	Elongated to rounded shape, occluded by individual hymenes	Areolae on rapheless valve round or transapically elongated externally, apically elongated internally	Not resolved in LM; elongated to rounded shape; occluded by individual hymenes	Uniseriate	N.d.	Not discernible in LM; small, rounded, becoming larger towards valve margins covered by cribrate structures internally
**Raphe valve**							
Raphe	Straight, drop-shaped central raphe pores; central nodule is unidirectional on outer side but turns to different side on the inner side; terminal raphe endings deflected and turn to opposite side	Straight, drop-shaped proximal endings; distal endings are deflected and turned in opposite directions and terminate at the edge of the valve margin	Straight, deflected to opposite sides near the apices; terminal raphe fissures strongly curved to opposite sides; external proximal raphe ends are simple, located in slight “pinhole” depressions; central raphe ends curve toward opposite sides internally	Straight, drop-shaped proximal raphe endings	Straight, tear-drop-shaped central raphe pores accompanied by ridges and grooves and turn in the opposite direction internally	Straight; the terminal fissures appear to curve in opposite directions; the proximal raphe ends are expanded in the shape of teardrops	Straight with straight, clearly expanded proximal raphe endings; distal raphe fissures deflected to opposite sides, terminating in droplike pores, almost invisible in LM
Axial area	Very narrow, linear, opening rather abruptly to the central area; raised on the inner side	Very narrow, linear, opening rather abruptly to the central area	Narrow, slightly sigmoid	Very narrow, linear, opening rather abruptly to the central area	Helictoglossae raised and turn to opposite directions	Narrow, widens abruptly into a transverse fascia	Narrow, opening rather abruptly to the central area; terminating on small helictoglossae
Central area	Raised central stauros on the inner side; bow-tie-shaped	Roughly symmetrical, narrow, rectangular-to-wedge-shaped fascia; shortened striae area are absent; forming a raised stauros on the internal side	Distinct fascia, often slightly wider on one side	More or less symmetrical, narrow, rectangular-to wedge-shaped fascia reaching the valve margins; shortened striae in the central area absent; forming a raised stauros internally	Wide, slightly asymmetric, bow-tie-shaped stauros	Bow-tie-shaped, occasionally bordered by a few shortened striae	Rather broad, rectangular, weakly asymmetrical fascia, lacking any shortened striae bordering the central area
Density of striae (/10 μm)	35–40	25–28	24–34	26.0–32.5	30–35	30–32	28–30
Striae	Uniseriate	At the valve ends, striae sometimes become biseriate near the axial area	N.d.	Uniseriate, almost parallel, becoming more radiate toward the apices	Striae near the central stauros are shorter than the other striae	N.d.	Narrower than the virgae, uniseriate
Density of areolae (/10 μm)	50–60	65–70	N.d.	70	N.d.	N.d.	N.d.
**Rapheless valve**							
Axial area	Lanceolate, fusiform, narrow	Lanceolate, narrows; turn slightly in the opposite direction at the end	Narrow and slightly sigmoid	Slightly raised internally above the surface	Sigmoid	Wider and expands gradually to merge with an asymmetric, irregular transverse central area	Narrow, opening rather abruptly to the central area
Density of striae (/10 μm)	15–22.5	25–28	20–25	25–30	30–35	24–28	23–24
Striae	Most uniseriate, become irregularly biseriate near the central and apices area	Most uniseriate, become irregularly biseriate near the central and apices area	N.d.	N.d.	N.d.	N.d.	Uniseriate; striae occasionally biseriate near the apices
Density of areolae (/10 μm)	40–50	65–70	N.d.	70	N.d.	N.d.	N.d.
Central area	Asymmetric, one side has one more stria than the other side; rectangular; irregular markings sometimes present in the central area	Asymmetrical wedge-shaped, on one side formed by shortened striae; slightly raised above the surface internally	Small, transapically rectangular, often asymmetric	Asymmetrical wedge-shaped, on one side formed by shortened striae; slightly raised above the surface internally	A reduced central area, with one or two lines of short striae on one side of stauros	N.d.	Narrow, rectangular, asymmetrical fascia, lacking any shortened striae bordering the central area; irregular markings (shallow depressions) sometimes present in the central area
References	This study	This study	[20,21]	[4]	[4,5]	[4,21,22]	[21]

**Table 2:** Estimates of evolutionary divergence among 20 closely related *Gogorevia* species based on *SSU* rRNA (1676 bp).

	Species	Strains	1	2	3	4	5	6	7	8	9	10	11	12	13	14	15	16	17	18	19	20
1	*Gogorevia constricta*	PQ046509		0.009	0.010	0.010	0.010	0.010	0.010	0.012	0.010	0.010	0.013	0.012	0.013	0.014	0.011	0.012	0.012	0.010	0.012	0.010
2	*Gogorevia recticentralis*	PQ034673	0.033		0.009	0.009	0.009	0.009	0.009	0.011	0.010	0.010	0.013	0.012	0.013	0.014	0.012	0.013	0.014	0.010	0.013	0.010
3	*Gogorevia uniseriata*	KY354249	0.045	0.033		0.002	0.005	0.003	0.011	0.012	0.011	0.011	0.013	0.012	0.012	0.013	0.013	0.012	0.014	0.011	0.012	0.010
4	*Gogorevia uniseriata*	MT193501	0.048	0.036	0.002		0.004	0.002	0.010	0.013	0.011	0.011	0.012	0.012	0.011	0.013	0.012	0.012	0.014	0.011	0.012	0.010
5	*Gogorevia rinatii*	MT193493	0.046	0.033	0.009	0.007		0.005	0.010	0.012	0.010	0.010	0.012	0.011	0.012	0.013	0.012	0.012	0.014	0.010	0.012	0.009
6	*Gogorevia ovalis*	MT193498	0.050	0.038	0.005	0.002	0.009		0.010	0.013	0.010	0.011	0.012	0.012	0.011	0.013	0.013	0.011	0.014	0.011	0.011	0.009
7	*Achnanthes chlidanos*	KJ658412	0.038	0.036	0.045	0.043	0.038	0.041		0.009	0.005	0.006	0.011	0.008	0.011	0.011	0.009	0.008	0.011	0.006	0.008	0.003
8	*Lemnicola hungarica*	KY354248	0.060	0.048	0.060	0.063	0.055	0.065	0.038		0.010	0.010	0.013	0.012	0.012	0.013	0.012	0.011	0.014	0.010	0.011	0.009
9	*Achnanthidium reimeri*	KJ658405	0.041	0.041	0.050	0.048	0.043	0.045	0.012	0.045		0.002	0.011	0.009	0.010	0.011	0.009	0.009	0.011	0.002	0.009	0.005
10	*Achnanthidium rivulare*	KJ658406	0.043	0.043	0.053	0.050	0.046	0.048	0.014	0.048	0.002		0.011	0.009	0.011	0.012	0.009	0.008	0.011	0.000	0.008	0.006
11	*Planothidium naradoense*	KY65079	0.076	0.071	0.068	0.065	0.066	0.065	0.055	0.076	0.055	0.058		0.012	0.004	0.008	0.012	0.011	0.013	0.011	0.011	0.011
12	*Achnanthidium minutissimum*	MH826401	0.055	0.063	0.065	0.063	0.058	0.060	0.031	0.060	0.038	0.036	0.065		0.012	0.012	0.010	0.008	0.012	0.009	0.008	0.008
13	*Planothidium frequentissimum*	KY650786	0.071	0.071	0.060	0.058	0.058	0.058	0.055	0.073	0.050	0.053	0.009	0.060		0.008	0.012	0.011	0.013	0.011	0.011	0.010
14	*Planothidium caputium*	KY650788	0.086	0.073	0.071	0.068	0.068	0.068	0.055	0.076	0.058	0.060	0.029	0.070	0.028		0.013	0.011	0.014	0.012	0.011	0.011
15	*Chamaepinnularia krookii*	OX386233	0.055	0.060	0.068	0.065	0.063	0.068	0.038	0.066	0.038	0.041	0.065	0.046	0.063	0.076		0.009	0.010	0.009	0.009	0.009
16	*Achnanthidium saprophilum*	ON040639	0.058	0.068	0.060	0.058	0.058	0.055	0.036	0.053	0.036	0.033	0.055	0.026	0.053	0.060	0.041		0.012	0.008	0.000	0.008
17	*Sellaphora pupula*	EF151974	0.065	0.083	0.086	0.083	0.081	0.086	0.053	0.083	0.053	0.055	0.081	0.066	0.078	0.091	0.043	0.063		0.011	0.012	0.011
18	*Achnanthidium kranzii*	KJ658397	0.043	0.043	0.053	0.050	0.046	0.048	0.014	0.048	0.002	0.000	0.058	0.036	0.053	0.060	0.041	0.033	0.055		0.008	0.006
19	*Achnanthidium catenatum*	MN602034	0.058	0.068	0.060	0.058	0.058	0.055	0.036	0.053	0.036	0.033	0.055	0.026	0.053	0.060	0.041	0.000	0.063	0.033		0.008
20	*Achnanthidium daonense*	KJ658413	0.038	0.041	0.041	0.038	0.033	0.036	0.005	0.038	0.012	0.014	0.058	0.028	0.050	0.055	0.038	0.033	0.053	0.014	0.033	

**Table 3:** Estimates of evolutionary divergence among 20 closely related *Gogorevia* species based on *rbcL* (705 bp).

	Species	Strains	1	2	3	4	5	6	7	8	9	10	11	12	13	14	15	16	17	18	19	20
1	*Gogorevia constricta*	PQ040372		0.007	0.013	0.012	0.012	0.011	0.010	0.011	0.012	0.012	0.012	0.012	0.013	0.013	0.012	0.013	0.012	0.013	0.012	0.013
2	*Gogorevia recticentralis*	PQ044338	0.031		0.012	0.012	0.011	0.011	0.010	0.011	0.012	0.011	0.012	0.011	0.013	0.013	0.012	0.013	0.012	0.012	0.012	0.013
3	*Gogorevia uniseriata*	MT210188	0.070	0.064		0.003	0.008	0.011	0.012	0.014	0.013	0.014	0.014	0.014	0.014	0.013	0.014	0.016	0.014	0.015	0.015	0.015
4	*Gogorevia uniseriata*	KY412433	0.066	0.068	0.004		0.007	0.011	0.011	0.014	0.013	0.014	0.014	0.015	0.014	0.012	0.014	0.016	0.014	0.015	0.015	0.014
5	*Gogorevia ovalis*	MT210186	0.062	0.060	0.033	0.029		0.010	0.011	0.014	0.013	0.014	0.015	0.014	0.014	0.013	0.013	0.015	0.014	0.016	0.014	0.014
6	*Gogorevia rinatii*	MT210180	0.061	0.064	0.058	0.054	0.056		0.010	0.013	0.012	0.013	0.014	0.013	0.013	0.012	0.012	0.014	0.013	0.015	0.014	0.013
7	*Lemnicola hungarica*	KJ658388	0.053	0.047	0.068	0.064	0.064	0.059		0.010	0.011	0.010	0.011	0.011	0.013	0.013	0.011	0.012	0.012	0.012	0.012	0.011
8	*Psammothidium papilio*	OX258990	0.068	0.072	0.089	0.084	0.091	0.084	0.059		0.010	0.004	0.008	0.010	0.012	0.012	0.014	0.013	0.012	0.010	0.012	0.011
9	*Planothidium naradoense*	KY650824	0.070	0.072	0.083	0.078	0.081	0.072	0.068	0.055		0.010	0.011	0.012	0.009	0.008	0.012	0.013	0.013	0.012	0.012	0.006
10	*Achnanthes chlidanos*	KJ658394	0.072	0.072	0.089	0.085	0.091	0.087	0.055	0.011	0.049		0.007	0.009	0.011	0.012	0.014	0.013	0.012	0.010	0.012	0.011
11	*Achnanthidium daonense*	KJ658395	0.078	0.078	0.093	0.098	0.104	0.095	0.068	0.031	0.063	0.027		0.009	0.013	0.012	0.014	0.014	0.012	0.010	0.013	0.012
12	*Achnanthidium saprophilum*	KY650833	0.074	0.057	0.091	0.095	0.091	0.084	0.065	0.049	0.068	0.045	0.045		0.013	0.013	0.014	0.014	0.013	0.007	0.013	0.012
13	*Planothidium* cf. *subantarcticum*	KY650822	0.078	0.072	0.089	0.085	0.089	0.089	0.078	0.070	0.047	0.063	0.080	0.074		0.007	0.014	0.013	0.012	0.012	0.013	0.010
14	*Planothidium lanceolatum*	KY650811	0.072	0.070	0.078	0.074	0.083	0.074	0.078	0.070	0.039	0.068	0.076	0.076	0.027		0.014	0.014	0.013	0.013	0.012	0.010
15	*Stauroneis schmidiae*	KM084967	0.070	0.074	0.089	0.085	0.083	0.078	0.063	0.089	0.074	0.086	0.086	0.089	0.087	0.084		0.011	0.012	0.013	0.012	0.012
16	*Halamphora turgida* var. *lacustris*	MG027502	0.082	0.072	0.106	0.102	0.095	0.093	0.076	0.082	0.076	0.078	0.093	0.084	0.078	0.087	0.057		0.011	0.013	0.011	0.013
17	*Amphora commutata*	KX120550	0.078	0.066	0.085	0.089	0.089	0.085	0.074	0.080	0.076	0.076	0.074	0.074	0.074	0.080	0.066	0.055		0.013	0.013	0.013
18	*Achnanthidium minutissimum*	KR709272	0.080	0.068	0.093	0.097	0.102	0.095	0.072	0.051	0.065	0.051	0.055	0.029	0.070	0.076	0.082	0.080	0.076		0.013	0.012
19	*Gomphonema parvulum*	JQ354690	0.074	0.072	0.095	0.091	0.091	0.089	0.078	0.074	0.074	0.074	0.091	0.080	0.074	0.076	0.068	0.061	0.074	0.076		0.012
20	*Planothidium caputium*	KY650806	0.080	0.076	0.091	0.087	0.091	0.082	0.068	0.065	0.017	0.059	0.074	0.070	0.053	0.051	0.080	0.078	0.083	0.065	0.072	

**Table 4:** Environmental and genetic data of *Gogorevia* collected from South Korea.

Species	Strain Number	Sample Locality	Collection of Date	Coordinates	Temperature (°C)	pH	DO (mg/L)	Conductivity (µs/cm)	Turbidity (NTU)	GenBank A.N. (*rbcL*)	GenBank A.N. (*SSU*)	KCTC A.N.
*Gogorevia contracta*	HYU-D119	Yangjaecheon(S1)	31 January 2024	37°29′13.56″ N, 127°3′20.84″ E	10.69	7.66	13.26	478	7.0	PQ040372	PQ046509	AG61355
	HYU-D120	Yangjaecheon(S1)	31 January 2024	37°29′13.56″ N, 127°3′20.84″ E	10.69	7.66	13.26	478	7.0	PQ040373	PQ046510	AG61356
	HYU-D121	Yangjaecheon(S1)	31 January 2024	37°29′13.56″ N, 127°3′20.84″ E	10.69	7.66	13.26	478	7.0	PQ040374	PQ046511	AG61357
*Gogorevia recticentralis*	HYU-D123	Godugyo(S2)	9 August 2023	37°51′16.04″ N, 127°44′52.09″ E	23.74	7.25	9.42	156	10.5	PQ044338	PQ034673	AG61359
	HYU-D125	Godugyo(S2)	9 August 2023	37°51′16.04″ N, 127°44′52.09″ E	23.74	7.25	9.42	156	10.5	PQ178746	PQ158273	AG61361
	HYU-D126	Godugyo(S2)	9 August 2023	37°51′16.04″ N, 127°44′52.09″ E	23.74	7.25	9.42	156	10.5	PQ178747	PQ158274	AG61362

A.N. = Accession number.

## Data Availability

The data supporting the findings of this study are available in NCBI GenBank.

## Supplementary Materials

The following supporting information can be downloaded at https://www.mdpi.com/article/10.3390/plants14091272/s1, Figure S1: Partial 18s rRNA transcript of *Gogorevia_contracta* sp. nov. (PQ046509); Figure S2: Partial 18s rRNA transcript of *Gogorevia recticentralis* sp. nov. (PQ034673); Figure S3: Partial 18s rRNA transcript of *Gogorevia uniseriate* (MT193501); Figure S4: Partial 18s rRNA transcript of *Gogorevia ovalis* (MT193498); Figure S5: Partial 18s rRNA transcript of *Gogorevia rinatii* (MT193493); Figure S6. Molecular phylogenetic analysis using the maximum likelihood (ML) method based on concatenated rbcL and *SSU* rRNA gene sequences, showing the phylogenetic position of *Gogorevia* species and related diatoms.

## Abbreviations

The following abbreviations are used in this manuscript:

LM	Light Microscopy
SEM	Scanning Electron Microscopy
*SSU* rRNA	Small Subunit Ribosomal RNA
*rbcL*	Ribulose-1,5-bisphosphate carboxylase/oxygenase large subunit gene
PCR	Polymerase Chain Reaction
DO	Dissolved Oxygen
WT	Water Temperature
NTU	Nephelometric Turbidity Unit
NCBI	National Center for Biotechnology Information
KCTC	Korean Collection for Type Cultures
A.N.	Accession Number
*ITS*	Internal Transcribed Spacer
*COI*	Cytochrome c Oxidase subunit I
