# Supplementary figures and images for: Morphology and Phylogenetic Positions of Two Novel Gogorevia Species (Bacillariophyta) from the Han River, South Korea
Source: Plants (Basel). 2025 Apr 22;14(9):1272. doi: 10.3390/plants14091272 (PMC12073239; doi:10.3390/plants14091272)

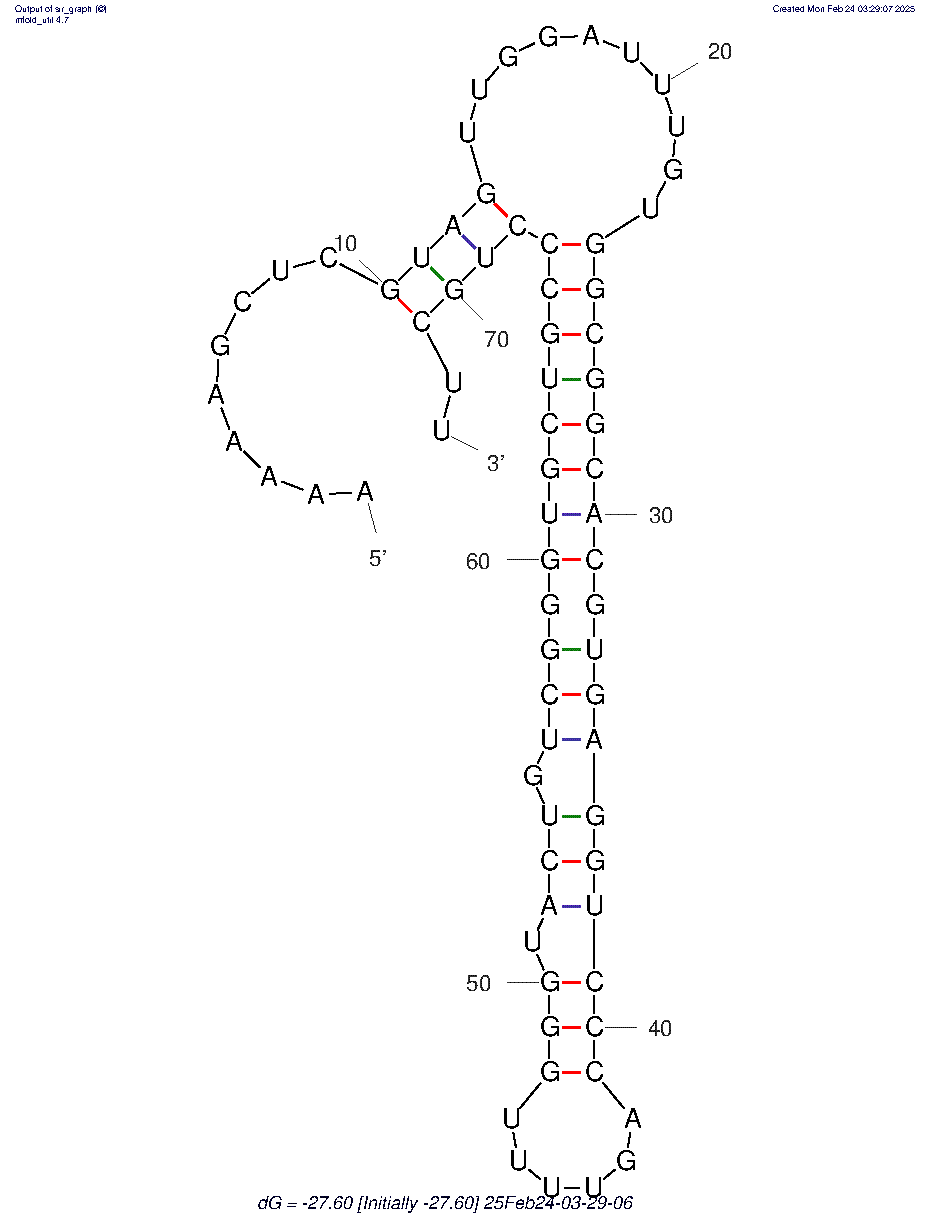

Supplement: Supplementary file 1 [file plants-14-01272-s001.zip › Figure S1 Partial 18s rRNA transcript of Gogorevia_contracta sp. nov. (PQ046509).png]

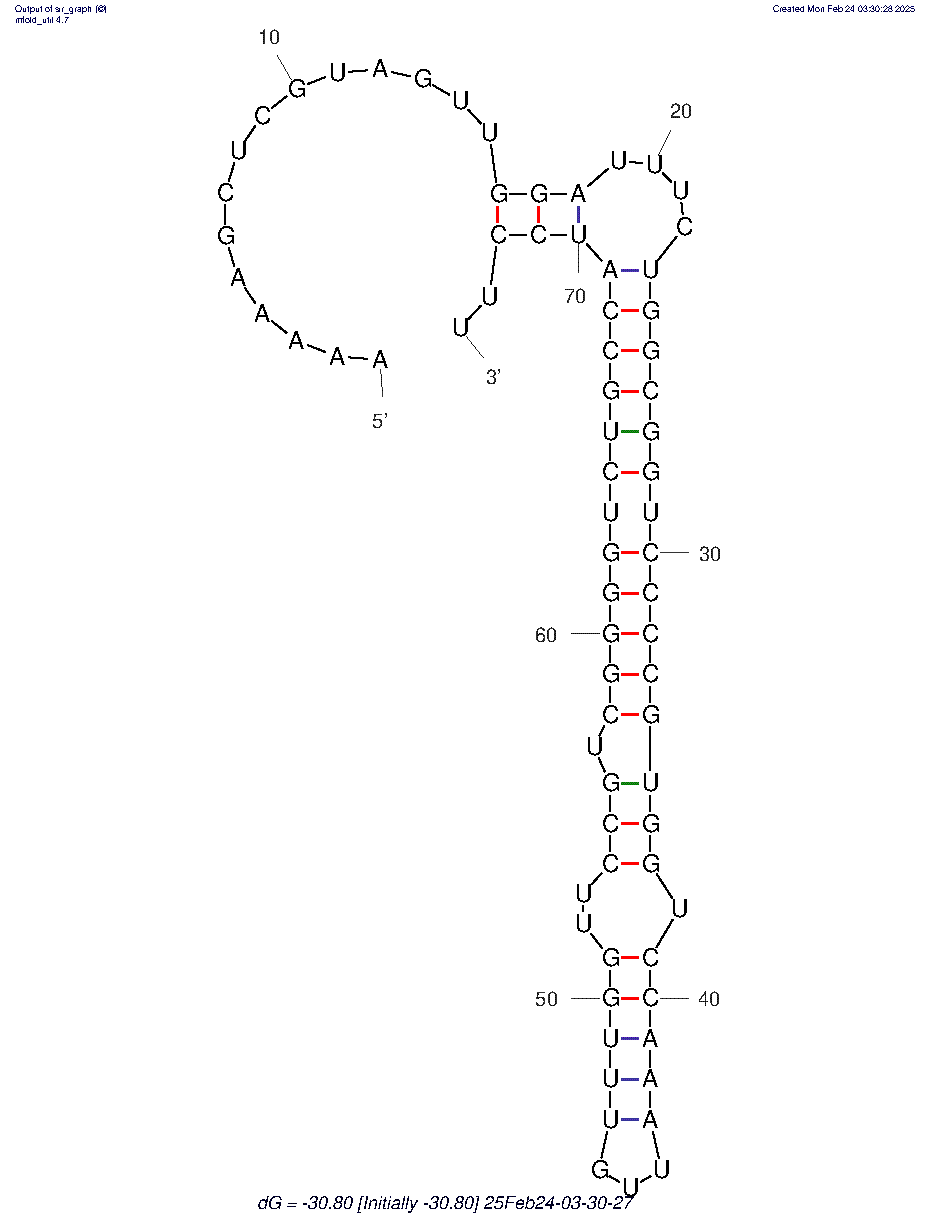

Supplement: Supplementary file 1 [file plants-14-01272-s001.zip › Figure S2 Partial 18s rRNA transcript of Gogorevia recticentralis sp. nov. (PQ046509).png]

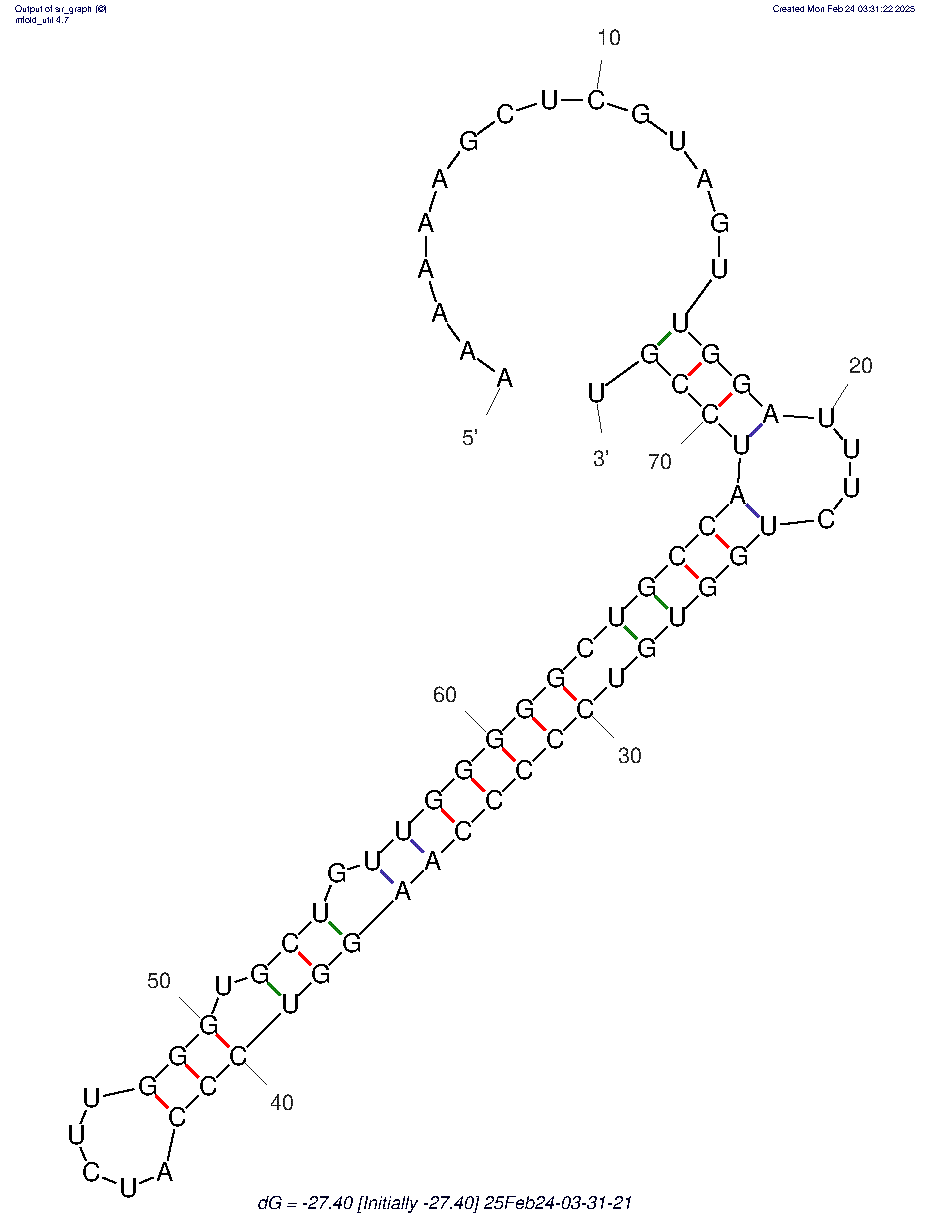

Supplement: Supplementary file 1 [file plants-14-01272-s001.zip › Figure S3 Partial 18s rRNA transcript of Gogorevia uniseriate (KY354249).png]

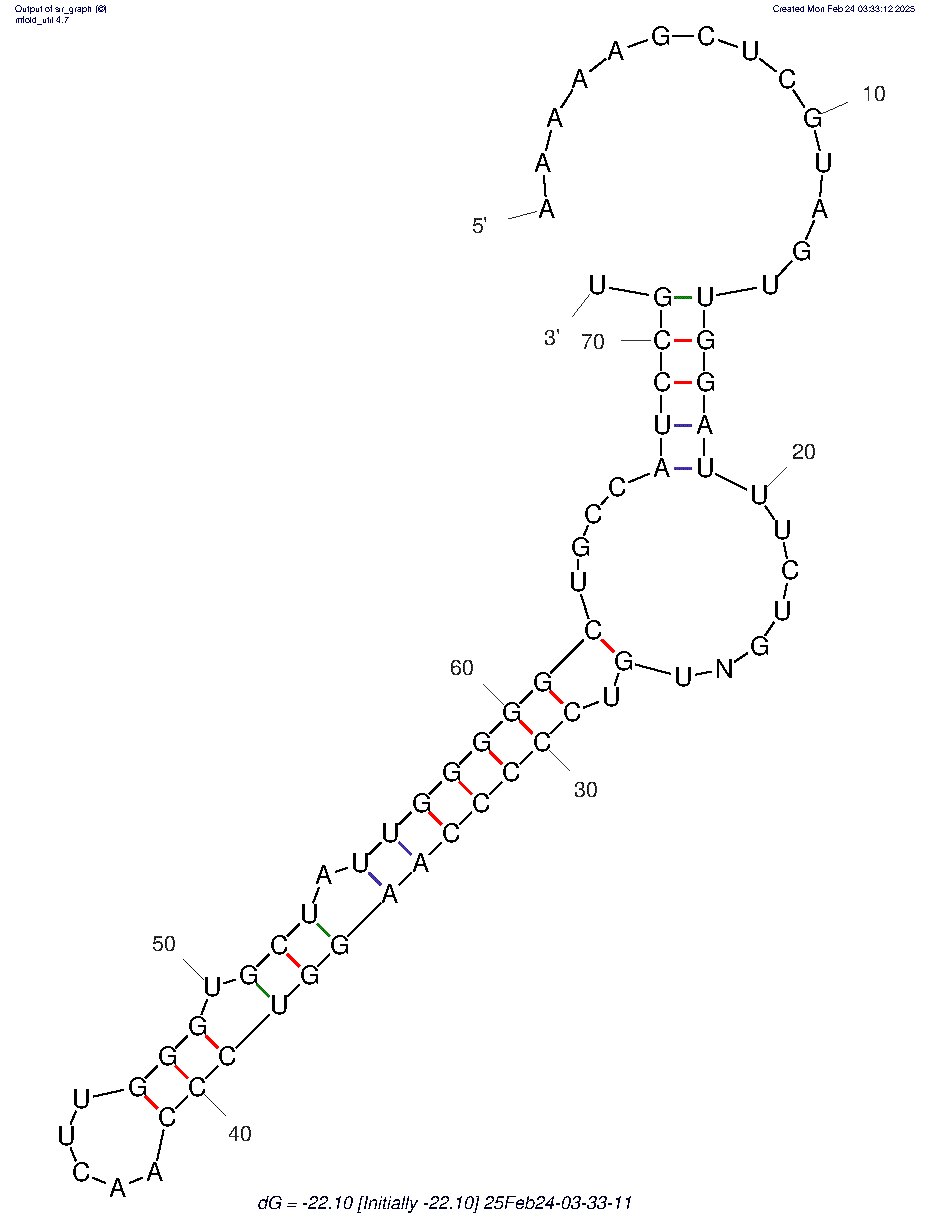

Supplement: Supplementary file 1 [file plants-14-01272-s001.zip › Figure S4 Partial 18s rRNA transcript of Gogorevia ovalis (KY354249).png]

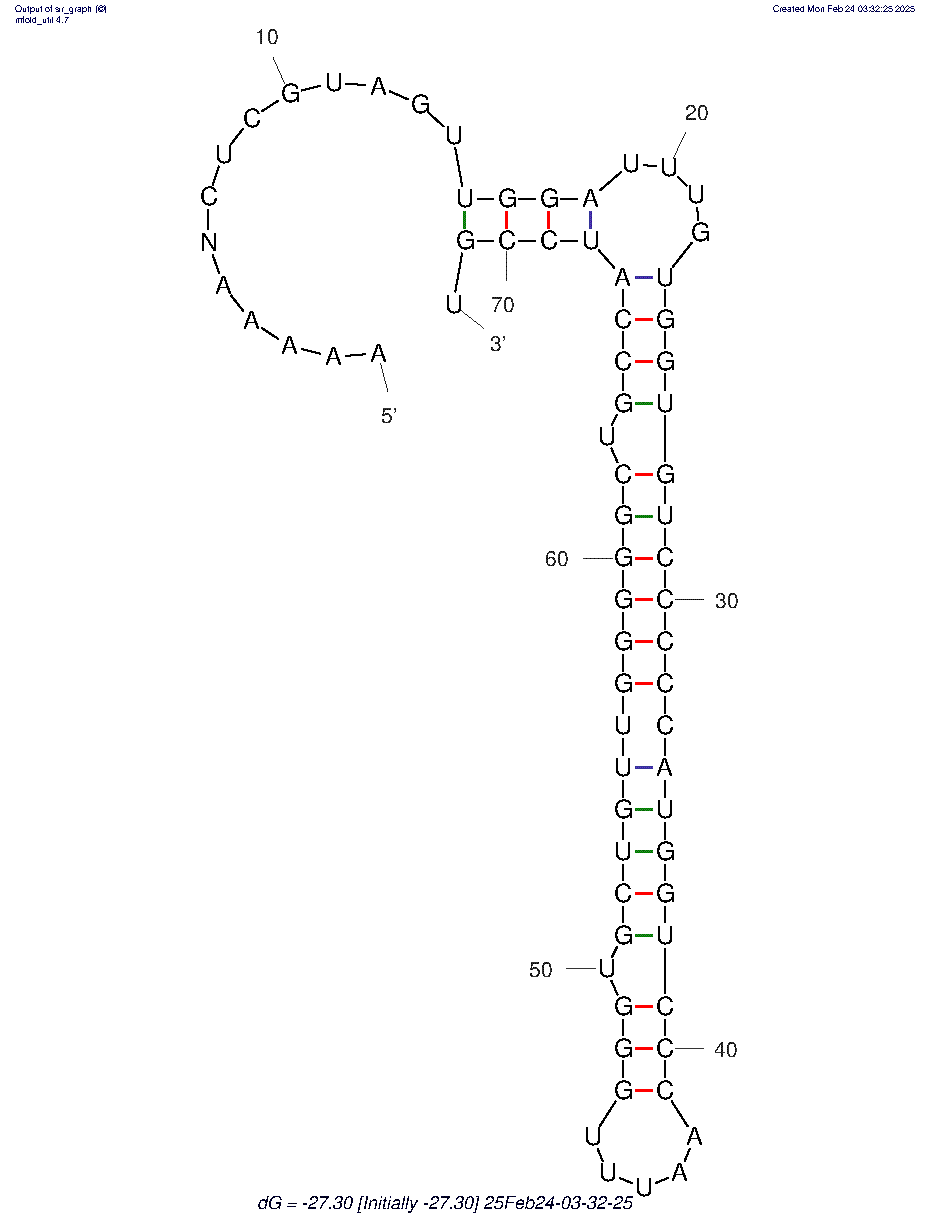

Supplement: Supplementary file 1 [file plants-14-01272-s001.zip › Figure S5 Partial 18s rRNA transcript of Gogorevia rinatii (PQ046509).png]

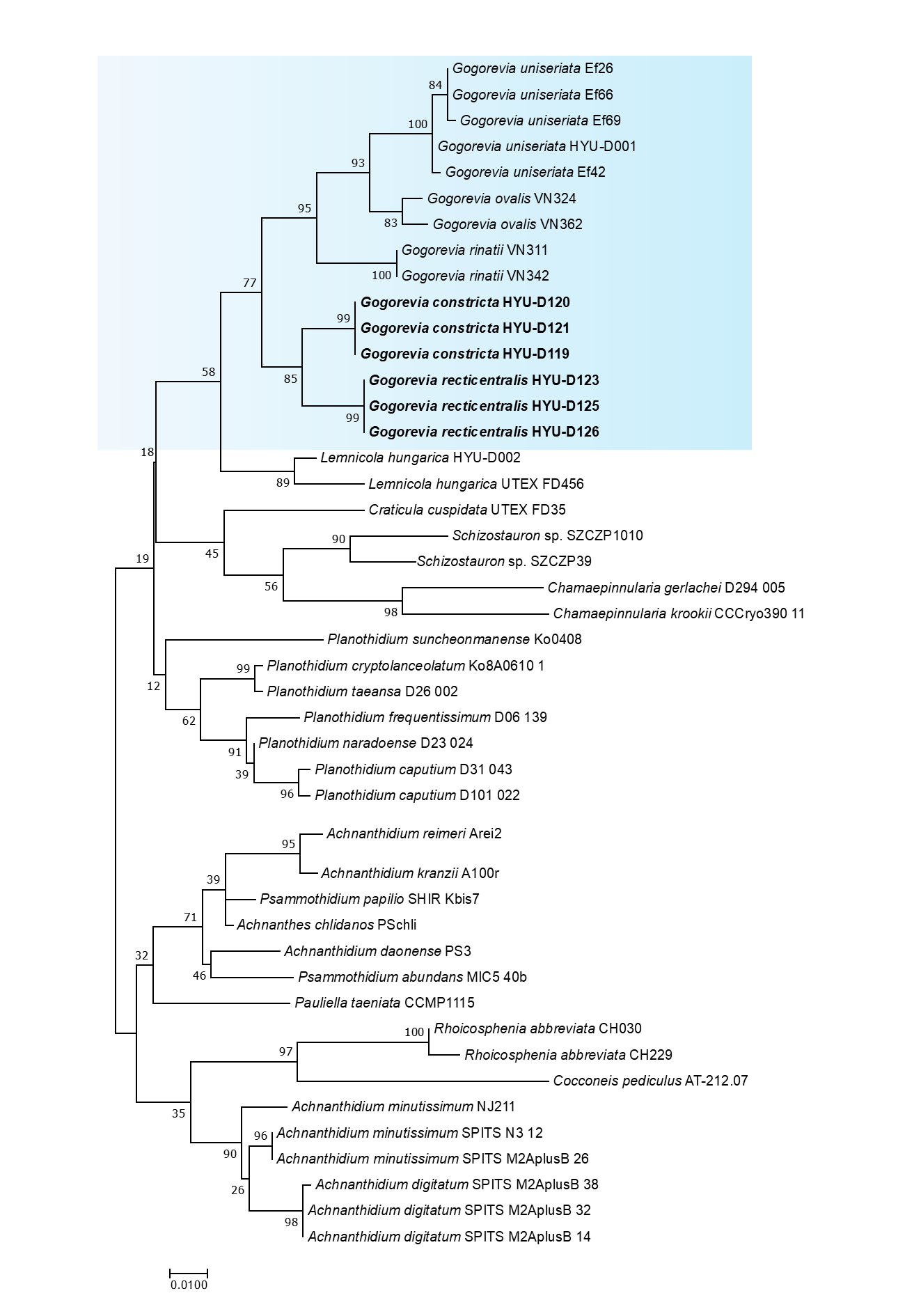

Supplement: Supplementary file 1 [file plants-14-01272-s001.zip › Figure S6 Molecular phylogenetic analysis using the maximum likelihood (ML) method based on concatenated rbcL and SSU rRNA gene sequences, showing the phylogenetic position of Gogorevia species and related diatoms..tif]
